# Primary Glaucoma in a Litter of Lop Rabbits

**DOI:** 10.1111/vop.70237

**Published:** 2026-07-25

**Authors:** Melaney A. Mayes, Cory M. Howard, Ellen K. Gratch, Jamie C. Morrison, Kelsey A. Brakel, András M. Komáromy

**Affiliations:** ^1^ Department of Small Animal Clinical Sciences, College of Veterinary Medicine Michigan State University East Lansing Michigan USA; ^2^ Veterinary Diagnostic Laboratory Michigan State University East Lansing Michigan USA; ^3^ Capital Area Humane Society Lansing Michigan USA

**Keywords:** angle closure, buphthalmos, goniodysgenesis, intraocular pressure (IOP), iridocorneal angle (ICA), pectinate ligament dysplasia (PLD)

## Abstract

**Objective:**

To describe pectinate ligament dysplasia (PLD) and primary glaucoma in a litter of adult Lop rabbits.

**Animals Studied:**

A litter of six adult Lop rabbits, three males and three females.

**Procedures:**

The animals were surrendered to the shelter. One male appeared functionally blind with bilateral severe corneal edema, and a second male showed severe corneal edema in one eye. Based on these observations, detailed ophthalmic examinations were performed on all six animals. Digital gonioscopy and fundoscopy were performed on three females. Because the two affected males were euthanized for humane reasons, and one female arrested during general anesthesia for ovariohysterectomy, six eyes of three animals were examined by routine histopathology.

**Results:**

Clinical examination revealed that the three eyes with diffuse corneal edema were affected by chronic glaucoma with intraocular pressure (IOP) elevation, buphthalmos, and severe secondary keratitis. Gonioscopy performed in the normal‐appearing, normotensive female rabbits showed partial or complete closure of the iridocorneal angle (ICA). Histopathologic evaluation revealed PLD in the glaucomatous and clinically normal eyes. In the glaucomatous eyes, uvea‐like tissue crossed the ICA with an indistinct to absent ciliary cleft, pectinate ligament, and scleral venous plexus. Inner‐to full‐thickness retinal atrophy was consistent with chronic glaucoma. In the non‐glaucomatous eyes, multiple segments of uveal tissue crossed the ICA, with the trabecular meshwork partially collapsed.

**Conclusions:**

This is the first formal description of PLD and primary glaucoma in a litter of Lop rabbits.

## 1 Introduction

Glaucoma is a group of degenerative optic nerve diseases that are leading causes of irreversible vision loss in humans and animals [1, 2, 3, 4]. In most human patients and all veterinary species, an increase in intraocular pressure (IOP) is the primary contributor to glaucomatous optic nerve degeneration [1, 2, 3, 4]. Primary glaucoma, in which increased IOP and retinal ganglion cell death occur without an identifiable preceding ocular disorder, is most commonly diagnosed in dogs; however, other animal species, including rabbits, can also be affected [5, 6].

Primary or hereditary glaucoma in New Zealand white rabbits and related breeds is the best‐characterized form of rabbit glaucoma [5, 6, 7]. This form of glaucoma appears to be autosomal recessively inherited, with not all presumed homozygous animals developing the disease, suggesting incomplete penetrance or a more complex pattern of inheritance [6, 8]. Although the *buphthalmos* or *bu* gene is frequently discussed as the underlying cause, neither the gene nor its encoded protein has been characterized, and genetic testing is not available [6, 8, 9]. Affected animals typically develop increased IOP (> 21 mmHg) and buphthalmos by 3–5 months of age [6, 7, 8, 10]. Gonioscopic and histopathologic evaluation of buphthalmic New Zealand white rabbits shows open iridocorneal angle (ICA) with absent or incompletely developed dysplastic pectinate ligament, solid sheets of uveal tissue spanning the ICA, agenesis of the ciliary cleft and trabecular meshwork [7, 8, 11].

There are only three original publications describing pet rabbits with glaucoma, including Lops and Lop crosses, New Zealand white, Lionhead, and Blanc de Hotot rabbits [12, 13, 14]. Because of chronic, end‐stage glaucoma with secondary pressure damage, the etiology could not be determined in most affected animals, but several affected rabbits showed histopathologic signs of primary glaucoma with goniodysgenesis [12, 13, 14]. More detailed information is needed on the prevalence and pathogenesis of primary glaucoma in pet rabbits. The purpose of this study was to provide the first detailed report of primary glaucoma in a litter of Lop rabbits.

## 2 Materials and Methods

Six rabbit littermates, males and females, roughly two years old, were surrendered to the animal shelter in two groups (Table 1). The first group consisted of three males, two of whom had clinically apparent ocular disease. The second group consisted of three clinically normal females. Of the six littermates, two were presumed albino based on iris and fur color, while the remaining four exhibited varying amounts of pigment.

**TABLE 1:** Summary of examination findings.

Case (coat color)	Sex	IOP: OD, OS (mmHg)	STT: OD, OS (mm/min)	Clinical diagnosis	Gonioscopy	Histopathologic diagnosis
Case 1 (white)	M	21, 27	8, 10	Chronic glaucoma with secondary ulcerative keratitis OU	NP	Closed ICA with PLD OU Segmental inner to full‐thickness retinal atrophy OU Ulcerative heterophilic and lymphoplasmacytic keratitis with subepithelial clefting and stromal fibrosis OU
Case 2 (white/brown or broken blue tort)	M	41, 15	6, 13	Chronic glaucoma OD Posterior polar cataract OS	NP	Closed ICA with PLD OD Segmental inner to full‐thickness retinal atrophy OD. Optic nerve atrophy OD Hyperplastic corneal epithelium with subepithelial clefting, stromal fibrosis, and segmental mineralization OD PLD and pigment‐laden macrophages OS Posterior cortical cataract OS
Case 3 (white/black or broken black)	M	16, 15	7, 11	Focal corneal scar OD Normal OS	NP	NP
Case 4 (white)	F	19, 19	8, 6	Posterior suture line cataracts OU	Partially closed ICA OU	PLD OU Mild central chromatolysis of retinal ganglion cells OD
Case 5 (white/brown or broken blue tort)	F	22, 20	7, 3	Normal OU	ICA closed OU	NP
Case 6 (white/black or broken black)	F	20, 21	9, 6	Normal OD Posterior polar cataract OS	ICA closed OU	NP

Abbreviations: F, female; ICA, iridocorneal angle; M, male; NP, not performed; OD, right eye; OS, left eye; OU, both eyes; PLD, pectinate ligament dysplasia; STT, Schirmer tear test.

All animals underwent an ophthalmic examination by the same board‐certified ophthalmologist and ophthalmology resident including slit lamp biomicroscopy (Kowa SL‐17; Kowa Company Ltd.), indirect ophthalmoscopy (Keeler All Pupil II headset; Keeler Instruments; 30D condensing lens; Volk Optical), rebound tonometry using the rabbit setting (Tono‐Vera Vet; Reichert Technologies), Schirmer tear test 1 (STT1; Color Bar Schirmer Tear Test; Katena Products Inc.), and fluorescein staining (I‐Glo Fluorescein Sodium Ophthalmic Strips; Jorgensen Laboratories LLC). Evaluation of visual performance testing was not possible due to inherent challenges when assessing a prey species. Reaction to light stimuli was assessed by dazzle and pupillary light reflexes using the brightest white light setting on the Kowa SL‐17 [5]. Vertical corneal diameter from dorsal to ventral limbus (12 to 6 o'clock position) was measured with Jameson calipers (Integra Miltex) to compare estimated globe sizes more objectively.

Routine ovariohysterectomy was performed on the three females under general anesthesia. The animals were premedicated with an intramuscular injection of buprenorphine (0.1 mg/kg; Endo Pharmaceuticals). General anesthesia was induced by intramuscular injection of ketamine (13 mg/kg; VetOne) and midazolam (1.75 mg/kg; Hikma Pharmaceuticals PLC). General anesthesia was maintained by an isoflurane/O_2_ gas mixture (1%–3% isoflurane; Piramal Pharma Limited) administered via a breather mask. Gonioscopy and fundus photography were performed under general anesthesia following surgery using the RetCam Shuttle with 130° lens (Clarity Medical Systems Inc.). Sterile, non‐ophthalmic ultrasound gel (Aquasonic 100; Parker Laboratories Inc.) was applied before the lens was placed on the cornea. Gonioscopy and fundus photography were not performed on the males because the equipment was unavailable when the animals presented to the shelter.

The two clinically affected males (Cases 1 and 2; Table 1) were euthanized for humane reasons with an overdose of intraperitoneal pentobarbital sodium (Fatal‐Plus; Vortech Pharmaceuticals Ltd), and the four globes were enucleated. One of the clinically normal females (Case 4) arrested under general anesthesia following ovariohysterectomy with unsuccessful resuscitation; both eyes (OU) were enucleated post mortem. The six enucleated eyes were fixed in 10% buffered formalin, and processed routinely for histopathologic examination following paraffin embedding, sectioning, and staining with hematoxylin and eosin (H&E) and Periodic acid–Schiff (PAS).

## 3 Results

### 3.1 Clinically Affected Rabbits (Cases 1 and 2)

Out of the six rabbits presented, two (Cases 1 and 2) had clinically apparent ocular disease (Table 1). Case 1 was bilaterally affected with buphthalmic globes and vertical corneal diameters of 18 mm OU (Figure 1A1,A2). Direct pupillary light and dazzle reflexes were present in the right eye (OD) and absent in the left eye (OS). The IOPs were 21 mmHg OD and 27 mmHg OS. Both corneas exhibited roughened topography, severe diffuse corneal edema, superficial neovascularization, and axial superficial ulceration with positive fluorescein staining. The severity of the corneal edema limited the evaluation of the intraocular structures, but the pupil appeared mydriatic OS and with little to no iris pigment OU. Limited view of the anterior chamber and lens did not reveal any overt opacities, and the posterior segment could not be visualized. The changes to the globes were consistent with chronic glaucoma and secondary exposure keratitis OU.

**FIGURE 1:**
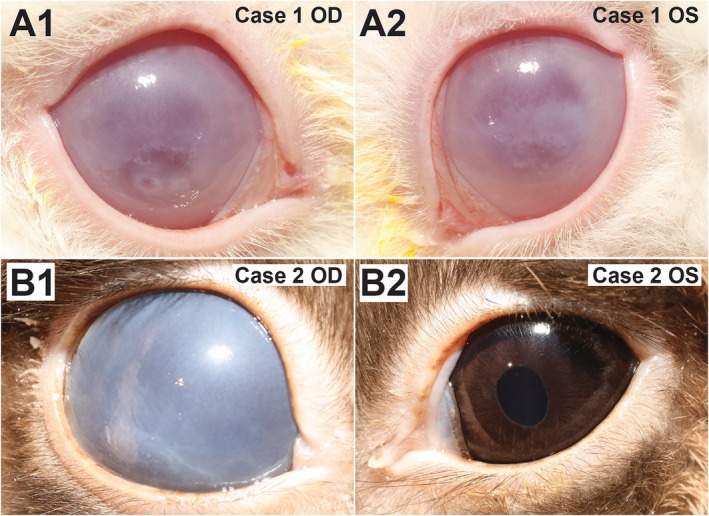
Eyes of the two glaucoma‐affected male Lop rabbits. (A) Case 1 was bilaterally affected. Both globes were buphthalmic. Both corneas exhibited roughened topography, severe diffuse corneal edema, superficial neovascularization, and axial superficial ulceration. (B) Case 2 was unilaterally affected. The OD was buphthalmic with severe diffuse corneal edema (B1). The OS was unremarkable (B2). OD, right eye; OS, left eye.

Case 2 presented ocular findings OD, including buphthalmia (vertical corneal diameter was 19 mm) (Figure 1B1). The IOPs were 41 mmHg OD and 15 mmHg OS. Examination of the anterior segment OD revealed severe diffuse corneal edema and a fixed, mydriatic pupil with no response to light (negative direct pupillary and dazzle reflexes). The fundus OD could not be visualized. The ophthalmic exam of OS was unremarkable, aside from an incipient posterior polar cataract; the vertical corneal diameter was 15 mm (Figure 1B2). Fluorescein staining was negative OU. The ophthalmic findings were consistent with chronic glaucoma OD.

### 3.2 Clinically Unaffected Rabbits (Cases 3, 4, 5, and 6)

Out of the six rabbits presented, four (Cases 3–6) had no clinical signs of significant ocular disease (Table 1). The ophthalmic examination on Case 3 was unremarkable OU, aside from a focal ventral paraxial corneal scar OD. Intraocular pressures were 16 mmHg OD and 15 mmHg OS. Fluorescein staining was negative OU, and both globes had positive direct pupillary light and dazzle reflexes.

Focal posterior suture line cataracts were present bilaterally in Case 4. Both optic nerve heads (ONHs) appeared normal (Figure 2A1,A2). Intraocular pressure was 19 mmHg OU. Fluorescein staining was negative OU, and both globes had positive direct pupillary light and dazzle reflexes. The ICA appeared partially closed on gonioscopy OU, and the pectinate ligament could not be visualized in some areas (Figure 3A1–A4).

**FIGURE 2:**
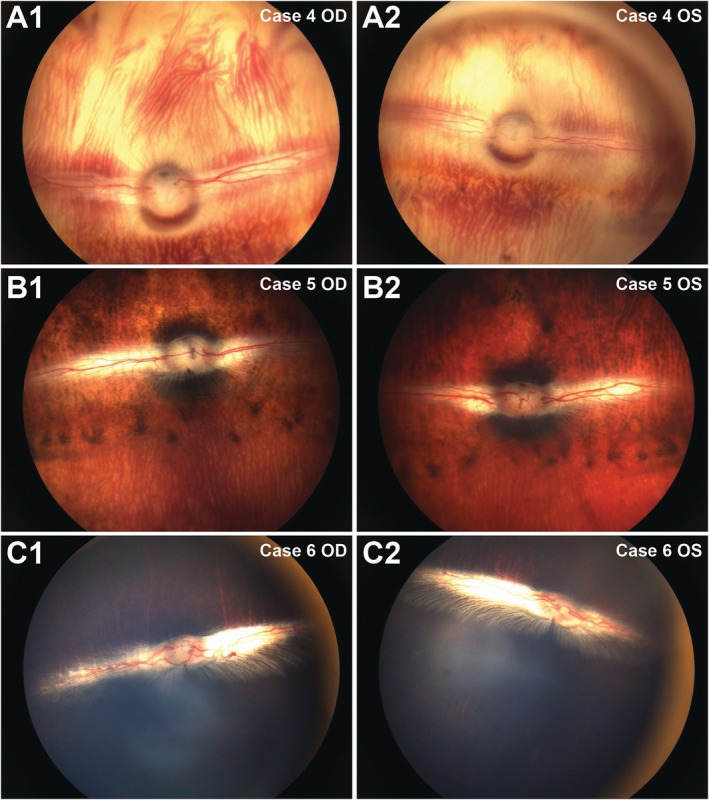
Fundus images of the three female non‐glaucomatous Lop rabbits. (A1, A2) Case 4 was an albino rabbit. Both ONHs appear normal with dark rims along the dorsal and ventral edges. These images were taken after the animal's cardiovascular arrest. (B1, B2) The fundus of Case 5 appeared normal and subalbinotic, consistent with the white/brown coat color. (C1, C2) The fundus of Case 6 appeared normal and well‐pigmented, consistent with the white/black coat color. OD, right eye; ONH, optic nerve head; OS, left eye.

**FIGURE 3:**
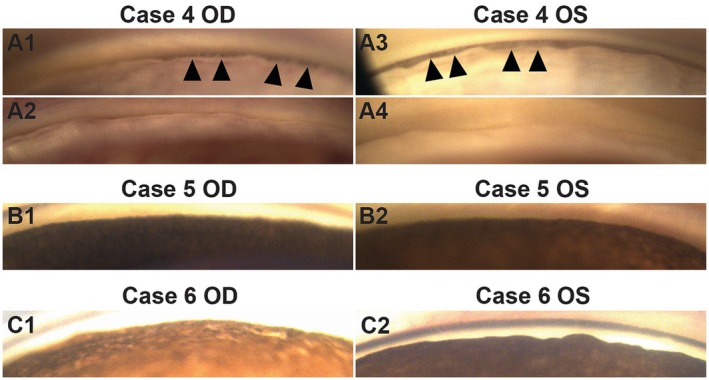
Gonioscopy of the three non‐glaucomatous female Lop rabbits. In case 4 (A1, A2—OD; A3, A4—OS), the images show regions with open ICA and normal appearing pectinate ligament (arrowheads in A1 and A3) and regions that appeared narrow or closed where the pectinate ligament could not be visualized (A2, A4). Gonioscopy of cases 5 (B1 and B2) and 6 (C1 and C2) only showed closed ICAs without visible pectinate ligament. However, the sharp contrast between the pigmented iris and the white sclera made it difficult to view the ICA opening. ICA, iridocorneal angle; OD, right eye; OS, left eye.

The ophthalmic exam on Case 5 was unremarkable with a normal subalbinotic appearing ocular fundus (Figure 2B1,B2). Intraocular pressures were 22 mmHg OD and 20 mmHg OS. Fluorescein staining was negative OU, and both globes had positive direct pupillary light and dazzle reflexes. The ICA appeared closed on gonioscopy, and the pectinate ligament could not be visualized (Figure 3B1,B2).

Case 6 had an unremarkable ophthalmic exam aside from an incipient posterior polar cataract OS. The ocular fundus appeared normal (Figure 2C1,C2). Intraocular pressures were 20 mmHg OD and 21 mmHg OS. Fluorescein staining was negative OU, and both globes had positive pupillary light and dazzle reflexes. The ICA appeared closed on gonioscopy, and the pectinate ligament could not be visualized (Figure 3C1,C2).

Corneal diameters were not measured on any of the clinically normal rabbits. Cases 3, 5, and 6 were adopted into new homes and were lost to follow‐up after the animals recovered from the neuter and spay surgeries.

### 3.3 Histopathology

Six eyes of three rabbits (Cases 1, 2, and 4) were submitted for histopathologic examination. Three eyes were buphthalmic and had clinical evidence of end‐stage glaucoma (Table 1). Overall, the severity of ICA abnormalities varied among cases and, in Case 2, between the glaucomatous and contralateral eye, with corresponding variation in the degree of retinal degeneration. For comparison, the histology of a normal rabbit ICA is provided (Figure S1).

*Case 1* Both albino eyes had ICA abnormalities consistent with PLD. Descemet's membrane tapered and blended into uvea‐like stroma that crossed the ICA. The ciliary cleft, pectinate ligament, and scleral venous plexus were indistinct (Figure 4A,C). These changes were interpreted as the cause of impaired aqueous humor outflow and glaucoma. There was variable ulcerative heterophilic and lymphoplasmacytic keratitis with subepithelial clefting and stromal fibrosis OU. Retinal changes differed between eyes: In OD, there was full‐thickness retinal atrophy with regionally extensive loss of the retinal pigment epithelium and adhesion of the remaining neuroretina to Bruch's membrane (Figure 4B). In OS, retinal atrophy was primarily confined to the inner retina characterized by retinal ganglion cell loss (Figure 4D).

**FIGURE 4:**
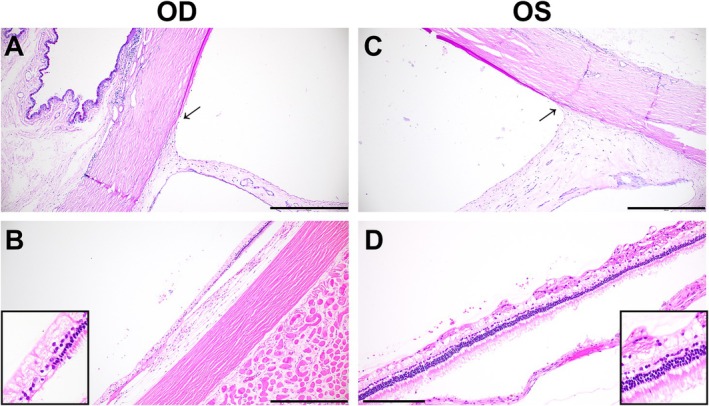
Histopathology of both glaucomatous eyes of Case 1. (A and C) Descemet's membrane tapers and blends into an imperforate uvea‐like stroma (→) crossing the ICA of OD and OS. The ciliary cleft, pectinate ligament (iris pillars), trabecular meshwork, and scleral venous plexus are inapparent. (B) Full‐thickness retinal atrophy is shown in OD. (D) OS shows segmental inner retinal atrophy. (A, C) PAS stain. Magnification = 100×. Scale bar = 500 μm. (B, D) H&E stain. (B) Magnification = 100×. Scale bar = 500 μm. (D) Magnification = 200×. Scale bar = 200 μm. Inset: higher‐magnification view, 400×, highlighting retinal detail. H&E, hematoxylin and eosin; ICA, iridocorneal angle; OD, right eye; OS, left eye; PAS, Periodic acid–Schiff.

*Case 2* The brown OD was glaucomatous and had similar ICA abnormalities as Case 1, including tapering of Descemet's membrane into uvea‐like stroma spanning the ICA, with indistinct ciliary cleft, pectinate ligament, and scleral venous plexus (Figure 5A). The corneal epithelium was hyperplastic with subepithelial clefts, and the superficial corneal stroma contained increased numbers of fibroblasts and segmental mineralization. Segmental inner‐ to full‐thickness retinal atrophy was present and was most severe ventrally (Figure 5B). The ONH was atrophic. The brown OS was clinically non‐glaucomatous but had mild ICA abnormalities consistent with PLD, including segments of uveal tissue crossing the ICA. The trabecular meshwork was partially collapsed, and the pectinate ligament was variably distinct (Figure 5C). Pigment‐laden macrophages were present in the ciliary cleft, and there was a focus of lens fiber degeneration in the posterior aspect of the lens cortex. The retina and ONH were histologically unremarkable (Figure 5D).

**FIGURE 5:**
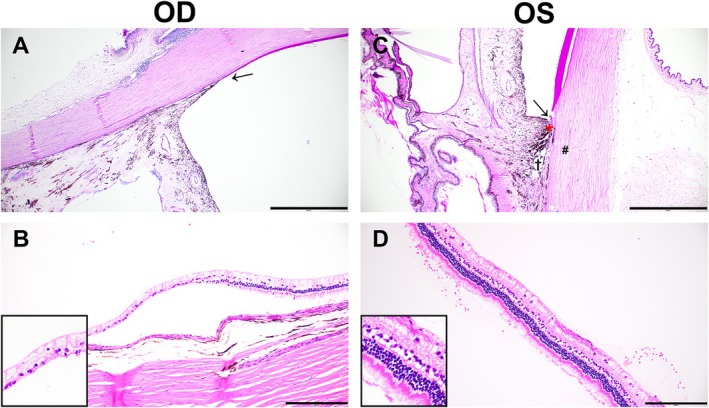
Histopathology of the glaucomatous (OD) and non‐glaucomatous (OS) eye of Case 2. (A) Descemet's membrane tapers and blends into an imperforate uvea‐like stroma crossing the ICA (→) OD. The ciliary cleft, pectinate ligament (iris pillars), trabecular meshwork, and the scleral venous plexus are inapparent. (B) The retina OD shows segmental inner‐to‐full‐thickness retinal atrophy. (C) Descemet's membrane inserts directly onto thick iris pillars (→), resulting in a partially collapsed ciliary cleft (*) and trabecular meshwork (†) OS. The scleral venous plexus is small (#). (D) No retinal atrophy is seen OS. (A, C) PAS stain. Magnification = 100×. Scale bar = 500 μm. (B, D) H&E stain. (B) Magnification = 100×. Scale bar = 500 μm. (D) Magnification = 200×. Scale bar = 200 μm. Inset: higher‐magnification view, 400×, highlighting retinal detail. H&E, hematoxylin and eosin; ICA, iridocorneal angle; OD, right eye; OS, left eye; PAS, Periodic acid–Schiff.

*Case 4* The examined albino eyes had relatively mild ICA abnormalities, reflected by a limited amount of uveal stromal tissue spanning the iridocorneal angle, a partially collapsed trabecular meshwork, and variably distinct pectinate ligaments (Figure 6A,C). These changes were considered consistent with mild pectinate ligament dysplasia; however, there was no histologic evidence of glaucoma aside from mild central chromatolysis of retinal ganglion cells in OD (Figure 6B). The retina and ONH were histologically unremarkable in OS (Figure 6D). This may be attributable to incomplete effacement of the ciliary cleft, with residual patency of the drainage angle allowing some continued aqueous humor outflow. Therefore, the histologic findings were not considered severe enough to support glaucoma in this case.

**FIGURE 6:**
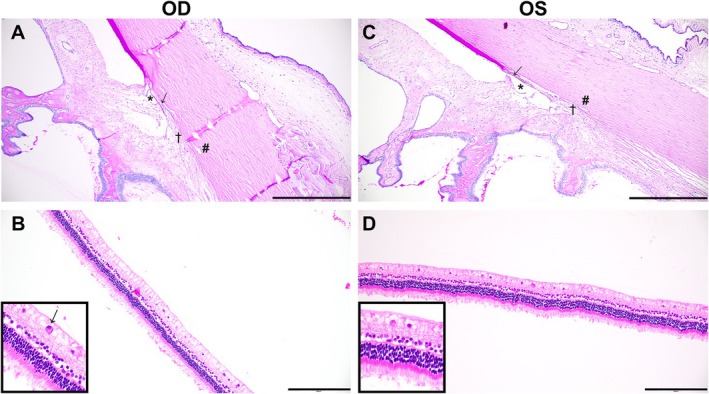
Histopathology of both non‐glaucomatous eyes of Case 4. (A and C) The ciliary clefts are partially filled OD and OS with loose, wispy mesenchymal tissue extending from the root of the iris (→). The ciliary cleft (*), iris pillars, trabecular meshwork (†), and scleral venous plexus (#) are present but variably distinct. (B and D) There is mild central chromatolysis of ganglion cells in OD (→). No retinal atrophy is seen in OS. (A, C) PAS stain. Magnification = 100×. Scale bar = 500 μm. (B, D) H&E stain. Magnification = 200×. Scale bar = 200 μm. Inset: higher‐magnification view, 400×, highlighting retinal detail. H&E, hematoxylin and eosin; ICA, iridocorneal angle; OD, right eye; OS, left eye; PAS, Periodic acid–Schiff.

## 4 Discussion

This study detailed clinical and histologic findings of glaucoma‐affected and glaucoma‐unaffected eyes, not only within the same litter but also within the same animal (Case 2). The diagnosis of primary glaucoma was made based on the presence of PLD in affected and related unaffected eyes and the absence of any other underlying disease process, such as uveitis. While presumed primary glaucoma in Lops has been described previously [12, 13, 14], this report represents the first detailed histological characterization of the disease. Future studies will need to confirm if the findings in this litter of Lops are representative of primary glaucoma in this breed. Chronically elevated IOP results not only in buphthalmos but also in secondary abnormalities within the ICA, which make it difficult to conclude the contribution of PLD in the pathogenesis. The presence of PLD in unaffected eyes in the same litter provided additional strong support for the conclusion that the Lop rabbits in this report were affected by primary glaucoma.

Rabbits with presumed primary glaucoma can present with bilateral or unilateral disease [7, 12, 13]; this was also the case in this litter of Lops, with one bilaterally (Case 1) and one unilaterally (Case 2) affected animal. The PLD found in the non‐glaucomatous eyes may predispose these unaffected eyes to glaucoma development in the future. Prospective studies are needed to better understand glaucoma risk and the age of disease onset in rabbit eyes with PLD. Concluding glaucoma risk from such studies can be challenging, as shown by comparable prospective studies in dogs with PLD [15, 16].

In this study, the IOPs in the glaucoma‐affected eyes ranged from 21 to 41 mmHg, compared to 15–21 mmHg in the unaffected eyes. Numerous studies have reported normal IOP in rabbits, ranging from 10 to 20 mmHg when measured by rebound tonometry [17, 18]. In rabbits with chronic glaucoma, the IOP may eventually return to normal, most likely due to ciliary body atrophy and reduced aqueous humor production [5, 12]. This may explain the relatively large IOP range in our glaucoma‐affected eyes and the small overlap with our unaffected IOPs.

The glaucomatous eyes of these Lop rabbits shared several similarities with the inherited end‐stage glaucoma in New Zealand white rabbits, most notably ICA abnormalities, such as uvea‐like tissue crossing the ICA and inapparent pectinate ligaments and ciliary clefts [7, 11]. It is possible that the Lop rabbits in this study were affected by the same genetic defect in the *bu* gene that causes primary glaucoma in New Zealand white rabbits [6, 8, 9]. Because the *bu* gene is not defined and genetic testing is not available, this etiology could not be investigated in this litter of Lop rabbits.

This study has several limitations, some of which are inherent to a case report. The findings represent a snapshot, with no information on the age of glaucoma onset or the likelihood that non‐glaucomatous eyes with PLD will develop glaucoma in the future. Not all clinical examination techniques were available for all six rabbits due to the diagnostics being performed at two separate visits. In addition, the ICA was evaluated only by gonioscopy, which can be impacted by pupil size, changes in IOP, and positioning of the goniolens on the cornea. High‐resolution ultrasound, optical coherence tomography, and electroretinography were unavailable [19]; however, detailed histopathologic data are provided. Because no inflammatory disease process was present in the glaucomatous eyes, testing for an *Encephalitozoon cuniculi* infection was not performed, even though this microsporidial parasite could cause secondary uveitic glaucoma [20]. In the absence of validated vision tests for rabbits, such as a reliable menace response [5, 20], assessment of vision was excluded from this report.

In conclusion, the presenting clinical and histopathologic findings in this small cohort of related Lop rabbits were consistent with primary glaucoma with PLD as a possible predisposing factor.

## Author Contributions

**Ellen K. Gratch:** investigation, methodology, writing – review and editing, conceptualization. **Jamie C. Morrison:** investigation, methodology, writing – review and editing, conceptualization. **Cory M. Howard:** investigation, writing – original draft, methodology, visualization, writing – review and editing. **András M. Komáromy:** conceptualization, investigation, funding acquisition, writing – original draft, methodology, visualization, writing – review and editing, supervision, resources. **Kelsey A. Brakel:** investigation, writing – original draft, methodology, visualization, writing – review and editing, supervision, resources. **Melaney A. Mayes:** conceptualization, investigation, writing – original draft, methodology, visualization, writing – review and editing.

## Funding

This work was supported by the National Institutes of Health (R01‐EY032478) and BrightFocus Foundation (G2022007S).

## Disclosure

The authors did not use AI to generate any part of the manuscript. AI tools were used to improve spelling, grammar, and general editing.

## Ethics Statement

This study adhered to the Guidelines for Ethical Research in Veterinary Ophthalmology (GERVO) and the Association for Research in Vision and Ophthalmology (ARVO) Statement for the Use of Animals in Ophthalmic and Vision Research.

## Conflicts of Interest

The authors declare no conflicts of interest.

## Supporting information


**Figure S1:** Histological structure of the ICA in a normal rabbit. (A) Descemet's membrane merges with the iris pillars arising from the iris root (→), posterior to which is the open ciliary cleft (*), trabecular meshwork (†), and intrascleral venous plexus (#). Periodic acid–Schiff (PAS) stain. Magnification = 100×. Scale bar = 500 μm. ICA, iridocorneal angle.

## Data Availability

The data that support the findings of this study are available from the corresponding author upon reasonable request.
