# Supplementary figures and images for: Primary Glaucoma in a Litter of Lop Rabbits
Source: Vet Ophthalmol. 2026 Jul 25;29(5):e70237. doi: 10.1111/vop.70237 (PMC13401460; doi:10.1111/vop.70237)

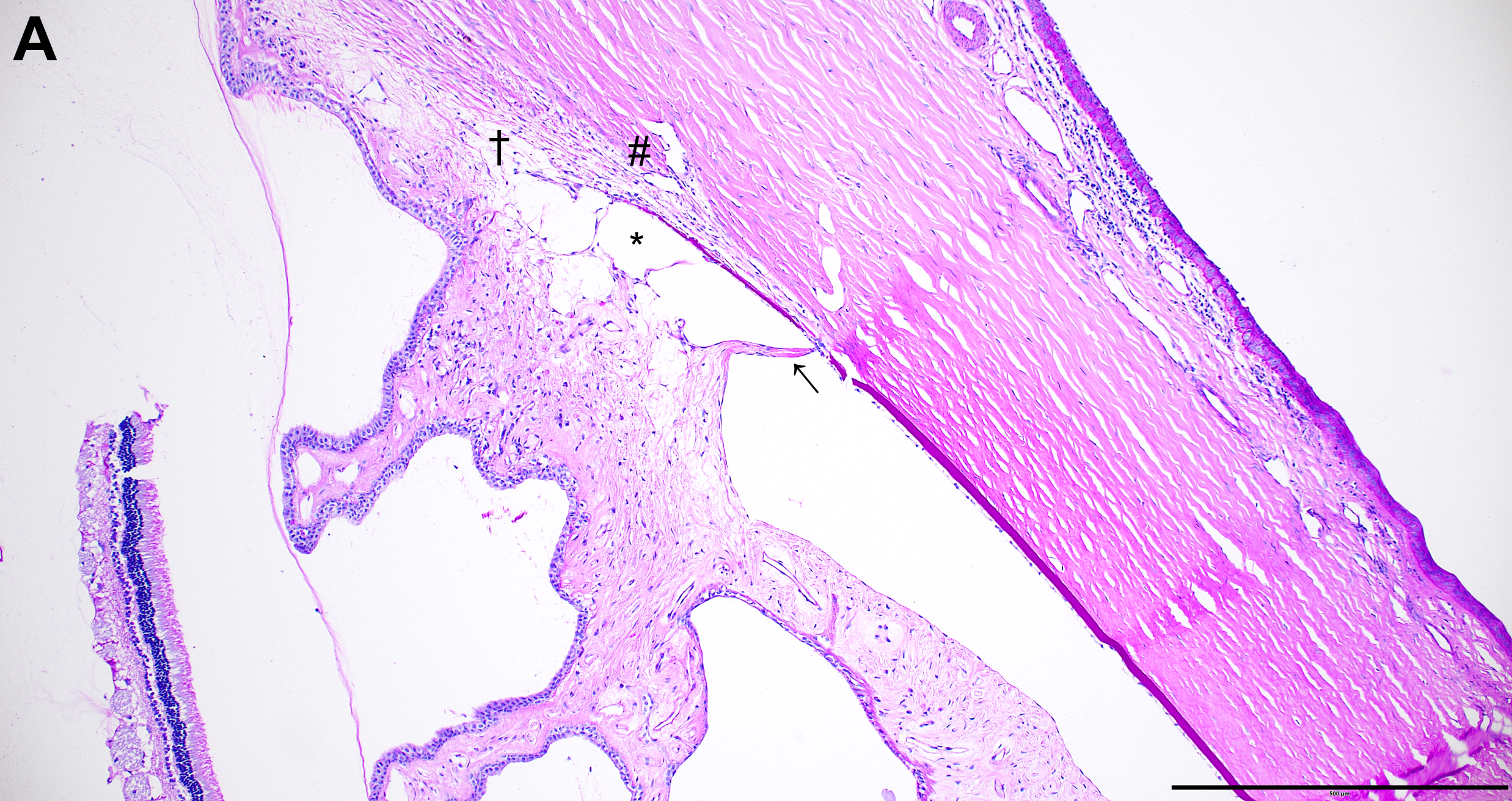

Supplement: Supplementary file 1 — Figure S1: Histological structure of the ICA in a normal rabbit. (A) Descemet's membrane merges with the iris pillars arising from the iris root (→), posterior to which is the open ciliary cleft (*), trabecular meshwork (†), and intrascleral venous plexus (#). Periodic acid–Schiff (PAS) stain. Magnification = 100×. Scale bar = 500 μm. ICA, iridocorneal angle. [file VOP-29-0-s001.png]
